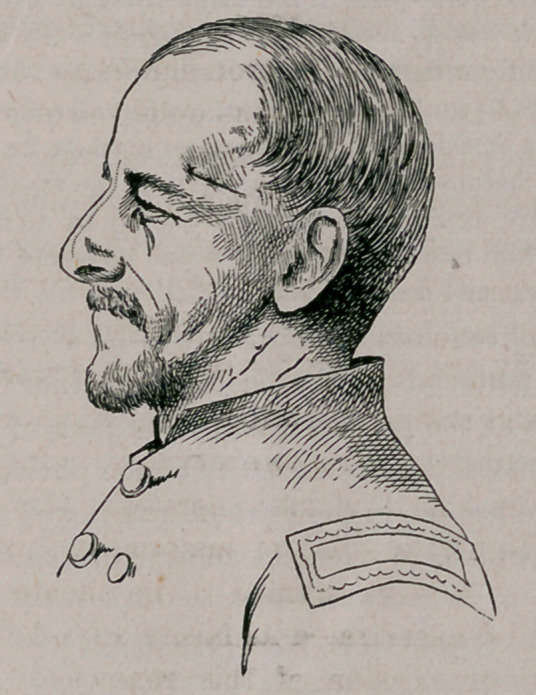# Phrenology

**Published:** 1887-06

**Authors:** 


### HALL'S JOURNAL OF HEALTH

TRUTH DEMANDS NO SACRIFICE; ERROR CAN MAKE NONE.

VOL. 34. JUNE, 1887. No. 6.

### SPECIAL.

HALL'S JOURNAL OF HEALTH will hereafter be supplied to newsdealers at the publication office, 206 Broadway, at wholesale rates.

We also offer, as a special premium to yearly subscribers, commencing with the July number and remitting one dollar, the previous numbers from January to June, 1887, inclusive.

An article by Dr. B. M. Lawrence, entitled "Bathing, its Agency in Therapeutics," also a translation of Dr. B. L. Cetlinski, entitled "Epistaxis," and another by Dr. Joseph Simms, on the "Physiognomy of the Nose," will appear in our July issue.

### PHRENOLOGY.

At the present day it is a matter of no little surprise, that the introduction of a system now so generally accepted as true, as that of *Phrenology* should have met with such determined resistance, from a class of minds always ready to condemn anything and everything, which oversteps the limits of their understanding, as if their malignant opposition could sweep the pioneers of progress from all new fields, and set bounds to the acquisition of knowledge.

Franz Joseph Gall, the founder of this interesting branch of science, which deals with the structure and functions of the brain, and their relation to the human system, was born in the duchy of Baden, in 1758, and, after having studied medicine at Vienna, settled there as a practicing physician, and became widely known by means of his published works upon medical and philosophical subjects, as early as 1791. Five years later, he began to deliver in public, lectures upon *Phrenology*, a term compounded of two Greek words, signifying a discourse on the mind, but his views were so *subversive of accepted doctrines* upon the same subject, as to arouse a spirit of opposition to him and his work, which gave rise to an edict of the Austrian government, in 1802, against their promulgation. Smarting under an injustice so ill-deserved, Dr. Gall, accompanied by his pupil, Dr. Spurzheim, who became his associate and shared in his fame, turned his back upon his adopted city, in 1805, and became a wanderer from city to city in other countries, wherein he presented his discoveries, and expounded his theories in a number of the leading universities, making some converts and stirring up no little opposition. Having settled in Paris in 1807, he there entered upon the practice of medicine, and continued to promulgate his discoveries, although discountenanced by Napoleon, who was quite averse to a foreigner's instructing the French in matters of science. A year later, Drs. Gall and Spurzheim presented a memoir of their discoveries to the Institute of France, upon which, a committee of that pretentious body, made an unfavorable report, to which Spurzheim replied in a volume treating, at length, of the brain and nervous system, which attracted wide-spread attention. Other volumes by Dr. Gall followed, which take rank among the most valued scientific works of his period, till now, all the leading features of his discoveries have come to be, upon every hand, admitted truths, and the name of their authors is held in due veneration. Having at one stage of his career, been publically accused of materialism and fatalism, Dr. Gall met the accusation in a work entitled "The innate order of Soul and Spirit," by which he demonstrated its falsity and uncharitableness.

But it is not in vindication of this renowned philosopher, whose standard was set up so far in the advance, nor of those labors which have made his name illustrious, that we are to speak. The necessity for the one is past, and for the other we have not sufficient skill. Our purpose is to offer a single element of proof, sustaining so far as it goes, the exactitude of his phrenological discoveries.

At the outbreak of our late civil war, one of the earliest to enroll himself on the Union side, was Lieut. Thomas W. Chandler, then a resident of Brooklyn. His enlistment was as a private in the 67th Regiment, New York Volunteers, afterwards attached to the army of the Potomac. Passing through the several grades of non-commissioned officers, to that of second Lieutenant, Chandler served with credit till honorably discharged from the service on account of a severe gun shot wound in the left temple, received in one of the later battles in which his regiment shared.

Prior to his enlistment, the occupation of Lieut. Chandler was that of a "cutter" for clothing houses, in which capacity he had acquired great expertness.

The profile likeness which we append, shows the locality of the wound, the bullet having fractured the skull, and become firmly wedged in the fracture. At his urgent request, Chandler was sent from the army hospital to Brooklyn, where he put himself in charge of the eminent surgeon, Dr. John G. Johnson, of that city, who, at our request, sent us the following account of this remarkable case, with the photograph from which the appended photo-engraving was made.

"At the time of my operation, the ball (an ounce and a quarter minnie with the butt twisted off like a flange), had been lodged in the brain for six weeks and one day. There was no loss of consciousness on the part of Lieut. C. He gave me a full and concise account of his case. There was no sensitiveness of the brain—about two tablespoonfuls of debris of brain material discharged out of the wound after removal of the ball—the trephine having been used to enlarge the opening to allow the ball to be removed. An abscess of that portion of the brain was forming, and on turning over the head this creamy, broken down brain debris run out of the wound. I am not prepared to say that any mental change took place, as the result of the injury to the brain. This portion of the brain is a terra incognita to physiologists. Ferrier says, in his work on the functions of the brain (in his experiments on monkeys and dogs) that "removal or destruction by the cautery of the antero-frontal lobes, is not followed by any definite physiological results. The animals retain their appetites and instincts, and are capable of exhibiting emotional feeling. The sensory faculties, sight, hearing, touch, taste and smell remain unimpaired. The powers of voluntary motion are retained in their integrity, and there is little to indicate the presence of such an extensive lesion or removal of so large a portion of the brain." They had, however, undergone a considerable psychological alteration. Instead of, as before, being actively interested in their surroundings, and curiously prying into all that came within the field of their observation, they became apathetic, or dull, or dozed off to sleep, responding only to the sensations or impressions of the moment, or varying their listlessness with restless and purposeless wanderings to and fro. While not actually deprived of intelligence, they had lost, to all appearance, the faculty of attentive and intelligent observation. P'g 254, on the Antero-Frontal region of the brain. This description of the destruction of this portion of the brain in animals, corresponded to what I observed in Lieut. Chandler's case. Before he went to the war he was rated as one of the best clothing cutters in the city, for instance, one of his old mates told me he was the only man in their house that could cut six dress coats at the same time, all the others cutting only a single one, yet his work was so accurate that the finest work in their large shop was assigned to him. And the wages that he received were by far, larger than any other man. After he returned to work, he seemed to have lost all snap and life in his business, his work had gradually deteriorated, so that after a while he was put only on ordinary shop work. He became morbid on account of the reprimands he received for his imperfect work. He would go to his work and try to do it, but failed, and when reprimanded, it made him morbid and reckless and he would then seem to feel that he was good for nothing, and lost all ambition."

By consulting a phrenological chart, it will be observed that the wound and loss of brain material in the instance of Lieut Chandler, who is now deceased, were at the point where the organs of *constructiveness* are located, thus demonstrating the accuracy of the phrenological deductions of Dr. Gall and his followers, in their investigations of the functions of the brain, the value of which, to science, can scarcely be overestimated.

## Figures and Tables

**Figure f1:**